# Information Needs of Pregnant Women in the COVID-19 Pandemic from Experts’ Point of View: A Qualitative Study

**DOI:** 10.30476/IJCBNM.2021.87447.1432

**Published:** 2021-04

**Authors:** Fatemeh Rezaei, Zahra Masaeli, Golrokh Atighechian

**Affiliations:** 1 Social Determinants of Health Research Center, Department of Health in Emergencies and Disasters, Isfahan University of Medical Sciences, Isfahan, Iran; 2 Community Health Research Center, Department of Nursing and Midwifery, The Islamic Azad University, Isfahan (Khorasgan), Iran; 3 Health Management and Economics Research Center, Department of Health in Emergencies and Disasters, Isfahan University of Medical Sciences, Isfahan, Iran

**Keywords:** COVID-19, Pandemics, Pregnant, Self-efficacy

## Abstract

**Background:**

As pregnant women are among vulnerable groups susceptible to respiratory infections, healthcare systems in most countries would pay more attention to providing the services required in the COVID-19 pandemic. The present study aims to identify the information needs of pregnant women during the COVID-19 pandemic.

**Methods:**

The research team conducted a qualitative conventional content analysis with an inductive approach to identify the views of 19 experts when working in the field of providing obstetric and midwifery services in Isfahan from April to June 2020. The semi-structured interviews were analyzed using MAXQDA v18.

**Results:**

The results showed that the information needs of pregnant women during the Covid-19 epidemic should be set in four areas, including 1) Self-efficacy of pregnant women, 2) Information that provokes sensitivity to the preventive measures, 3) Awareness of the perceived threat, and 4) Awareness of the health system functions in COVID-19 pandemic.

**Conclusion:**

The study shows that the self-efficacy of pregnant women depends deeply on being informed of the general and specific self-care principles. Besides, sensitivity could be achieved through the increased risk perception and knowledge on the pandemic. However, pregnant mothers should know the potential threats that could pose them at risk of vulnerability. Finally, awareness of the health system functions regarding access to reliable information resources along with provided services at Medical University websites is also recommended.

## INTRODUCTION

Human coronaviruses (COVID-19) and severe acute respiratory syndrome coronavirus (SARS-CoV) cause worldwide epidemics with high morbidity and mortality rates. No effective drug has been found in randomized controlled clinical trials to date. However, few drugs have been suggested and tested. According to the reports, 15.4% of pregnant women who presented for delivery were tested positive for SARS-CoV-2 by applying universal tests in all pregnant women. ^1 , 2^ Pregnant women are among the vulnerable groups more susceptible to viral respiratory infections and pneumonia due to physiological and immunological measurements. ^3 , 4^ Other strains of the COVID-19 virus, such as SARS, MERS (Middle East respiratory syndrome), and flu have higher mortality and morbidity rate in pregnant women than general people. The epidemics of SARS, MERS showed increased miscarriage and stillbirth, especially in the first trimester. ^5^ According to the Centers for Disease Control and Prevention (CDC) recommendations, pregnant women should be advised to prevent Covid-19 infection like other women. As the results of evidence-based research on Covid-19 have not yet been published, little scientific evidence is available on the side effects of the infection on pregnant women. Therefore, healthcare centers are responsible for taking adequate precautions to prevent exposure of pregnant women to infected patients. Meanwhile, pregnant personnel in hospitals are also at greater risk. ^6^

In affected countries, prevention and control measures for pregnant mothers were considered a major concern due to vertical transmission risk. However, despite the small number of pregnant cases, the nasopharyngeal swabs, amniotic fluid, umbilical cord, and breast milk were tested to assess the possibility of vertical transmission. Although previous studies showed that the risk of vertical transmission is low, there are still concerns about comorbidities for the fetus and mother due to the similarity of pathogenesis with SARS. ^7 , 8^ Therefore, pregnant women and infants are considered high-risk groups. Accordingly, the National Health Commission of China necessitates more sensitivity to continuous maternal care, screening of pregnant women, and follow-up preparations during timely visits. In addition, some studies emphasized isolating the infants with COVID-19 for at least 14 days and not breastfeeding to prevent close contact with the suspected or confirmed mothers. ^9 , 10^

When Iran reached the third peak of the pandemic, more than 1,040,547 people were affected, and more than 50,310 people died. At the time, schools, educational institutes, universities, and private sectors were closed. Besides, the Ministry of Health and Medical Education (MOHME) published useful national guidelines and instructions on environmental hygiene of public places and hospitals, medical staff precautions, principles of the use of personal protective equipment (PPEs), etc. However, recommendations on the principles of maternal-fetal care and services were few among the published documents. Besides, among published documents and data nationally and internationally, a high discrepancy was found regarding morbidity, mortality, medication, and vertical transmission in the pre-partum and postpartum COVID-19 infection in pregnant women. ^11 - 13^ Therefore, as there is not a reliable and credible knowledge resource for pregnant women, effects on their mental health would result in anxiety, depression, and high-stress levels. ^14 , 15^ A study has shown that most concerns of pregnant women were due to hospital visits (72.65%), protection procedures (60.17%), infant’s safety (52.14%), social media messages (40.68%), and contraction of the infection (39.83%). Regarding this, obstetricians propose the provision of more information resources such as videos, websites, and counseling services for perinatal women. ^16^ In addition, because the literacy level of pregnant mothers is not the same, transparent information with simplicity should be provided to give the required information. In this regard, as information needs might differ due to the safety culture, disaster preparedness training, and response capacity of the health system in a community, a qualitative study is required to extract information needs based on each context. Therefore, the present study aimed to look for the information needs of pregnant women during the COVID-19 epidemic from the experts’ perspectives to identify the necessary information to be compiled as guidelines for officials and specialists.

## METHODS

As the aim of this study was to identify and describe different attitudes toward the information needs of pregnant women in the Covid-19 pandemic, inductive conventional content analysis was used in this study. The research population included health professionals in the field of midwifery services, obstetricians, gynecologists, nurses, midwives working in the obstetrics and gynecology wards in (private or public) hospitals, personnel of Medical Universities in the field of midwifery services, policymakers, managers, and experts in the field of midwifery, and managers and midwifery experts in private and public midwifery clinics. The inclusion criteria were experts with relevant education and occupation in the fields of obstetrics and midwifery services. Experts with less than five years of experience in providing midwifery services and those who deliver healthcare only in their private offices were excluded. Purposive sampling was initially conducted, and then the snowball method was used to identify further participants. Regarding this, experienced participants with the necessary knowledge in the field of midwifery services and serving pregnant mothers in referral hospitals in epidemics were selected. Sampling continued until data saturation. In this study, 19 participants were interviewed.

Semi-structured interviews were conducted to collect data from January to April 2020 in Persian. Some interviews were administered at the workplace of the participants in a private meeting. However, other interviews were conducted through social media and distance interviewing to prevent transmission via close contact and follow quarantine restrictions. The research team used WhatsApp video call to hold ten distance interviews. Some interview questions were: What are the information needs of pregnant women with COVID-19 infection?, What are the information sources that pregnant women need to know?, and What are the healthcare services provided by the Medical Universities that pregnant women should be aware of? (For the COVID-19 infection). Besides, the interviewers’ memos were written during the interviews (for both video calls and face-to-face interviews) to analyze broader perspectives of experts on particular aspects of information needs based on the interviewers’ interpretation during data coding.

The research team provided an electronic informed consent form to be filled out by participants before starting the interviews. The interviews were recorded and transcribed verbatim. Each interview lasted for 30 to 60 min. The interview protocol was pilot tested with two midwives who were not part of the sample.

The transcripts were uploaded into MAXQDA 10 and coded by the first (FR) and second (ZM) authors. The research team then reﬁned the previous main categories through online-meetings and interviews. This means that units captured by both authors were coded and categorized while those captured just by one author were subject to further analysis. The meaning units were revised to support the overriding categories and subcategories. 

The data were analyzed in three phases, including preparation, organization, and reporting. First, the interviews were transcribed verbatim, followed by a repeated study of the text for data immersion (preparation). Second, the related meaning units and codes were grouped. Then, similar groups were categorized, and finally, the abstraction process was done to reach the main categories (organization). The final stage was reporting the extracted categories. The main categories were finalized after team discussions in multiple meetings (reporting). The research team reached 362 open codes sorted into 20 subcategories. Then, subcategories were merged into four main categories.

For evaluating the trustworthiness of the study data, four criteria were used in Lincoln and Guba, namely credibility, conformability, dependability, and transferability. In order to achieve credibility, various age groups and specialties (physicians, nurses, midwives, and specialists) were selected to identify different views and discover the concepts. Two independent specialists in qualitative studies checked the interview categories that emerged during analysis for confirmability, and inter-rater reliability was 92 percentage (dependability). Regarding transformability, the emergent themes returned to health professionals who had not been interviewed but met the inclusion criteria.

This study received the required ethics approval from Isfahan University of Medical Sciences Research Ethics Committee (IR.MUI.RESERCH.REC.1398.734), Isfahan, Iran. The anonymity of participants was assured by replacing the names with interviewees’ registration codes. 

## RESULTS

The participants were six obstetricians from the public sector and eight specialists from private hospitals. All interviewees were experts aged 45-54 years. Besides, most of them were holders of Master of Science and doctoral degrees (Table 1). 

**Table 1:** Demographic characteristics of the participants

Participant	Age (years)	Qualification	Occupation	Experience (Year)
1	48	MD^a^	Obstetrician	23
2	45	BS^b^	Midwife	13
3	49	MS^c^	Midwife	7
4	57	MD	Obstetrician	25
5	46	BS	Midwife	17
6	55	MS	Midwife	21
7	38	MS	Midwife	14
8	69	MD	Obstetrician	15
9	54	BS	Midwife	20
10	50	MD	Obstetrician	28
11	42	MS	Midwife	16
12	67	MS	Midwife	20
13	59	MS	Midwife	17
14	46	BS	Midwife	10
15	63	MD	Obstetrician	31
16	45	MD	Obstetrician	35
17	75	MS	Midwife	14
18	53	BS	Midwife	16
19	44	BS	Midwife	14

a Doctor of Medicine;

b Bachelor of science;

c Masters of science

We obtained four categories using content analysis. Table 2 shows the subcategories of each main category.

**Table 2:** Codes, sub-categories and categories regarding the information needs of pregnant women

Codes	Subcategories	Categories
Necessary daily activities during the quarantine period	General self-care principles	1. Self-efficacy of pregnant women
Healthy diet		
Preserving mental health at home		
Raising awareness about quarantine principles.		
Being careful when using disinfectants		
Improving knowledge about stability of maternal vital signs.	Specific self-care principles	
Monitoring pregnancy complications due to COVID-19 infection.		
Monitoring COVID-19 infection symptoms and relevant maternal comorbidities.		
Providing different information resources based on the literacy level of the mothers	Increasing the risk perception of pregnant mothers	2. Information that provokes sensitivity to the preventive measures
Reducing close contact with children		
Reducing contact with close relatives		
Patient-based safe actions	Being informed of the requirements of safe midwifery services	
Health provider safe actions		
Risk of vaccination for pregnant mothers	Basic information about the COVID-19 infection	
Epidemiology of the COVID-19 infection		
Contact risk	The threats posed to babies born to mothers with known COVID-19	3. Awareness of the perceived threat
Breastfeeding Risk		
Limitation to prescribing medicine for mothers with known COVID-19	Risk factor of Vulnerability	
Taking radiographs with a very low fetal radiation dose		
Pregnant women having chronic diseases		
Potential threats due to decreasing social trust	Social media threats	
Potential threats due to disseminating incorrect information		
Potential threats due to increasing fear and panic		
Awareness of access links of scientific information.	Being aware of information sources of Medical Universities	4. Awareness of the health system functions in COVID-19 pandemic
General information about COVID-19 on Universities’ websites		
Visual training by posters, pamphlets, videos, etc.		
Assurance of adequacy of health services	Being aware of the provided services	
Being aware of referral health centers for pregnant women		
Being aware of home-based maternal services		
Being aware of telemedicine services		

### 1. Self-efficacy of Pregnant Women

The self-efficacy of pregnant women is the category contained in 30% of the study codes. Self-efficacy contained two subcategories: general self-care principles and specific self-care principles.

General self-care principles help following the daily activities, healthy diet, preservation of mental health, quarantine principals at home, and careful disinfectants usage during the epidemic.

Regarding daily activities, one interviewee mentioned: 

*“Even healthy pregnant women should not have much contact with others.... they should avoid traveling, leaving home for hairdressing, shopping, and being in crowded centers....”* (P9)

Some interviewees emphasized making pregnant women aware of good nutrition procedures: 

*“Pregnant mothers should become aware of eating foods rich in protein such as ones containing meat ... they should know the importance of avoiding undercooked, grilled, and fried foods...and eat foods containing enough minerals, such as fruits and vegetables ….”* (P6).

One participant discussed the necessity of preserving mental health:

*“Pregnant women should avoid stress and anxiety. That is, continuous following of news concerning the number of deaths and confirmed/severe cases that cause anxiety should be limited.”* (P11).

Regarding quarantine principals at home, the interviewees explained important points such as:

*“…The information needs of pregnant women include the purpose, importance, requirements, and principles of care and disinfection for at-home quarantine…”* (P7).

One interviewee notified pregnant women of being careful when using disinfectants: 

*“Pregnant mothers should pay attention to the safety precautions when using disinfectants that are available to everyone”* (P4).

Specific self-care principles refer to self-monitoring the maternal vital signs, pregnancy complications, and COVID-19 infection symptoms during the quarantine period. Therefore, pregnant women can inform their doctors and midwives of severe symptoms wisely and on time. Besides, self-efficacy provokes sensitivity of pregnant women to the preventive measures to be discussed in the next category.

Regarding how to control the maternal vital signs at home, one interviewee explained important points such as: 

*“...Pregnant women should be aware that if they gain more than one kg weight in a week and observe significant changes in blood pressure, it’s essential to visit their healthcare providers”* (P5).

Pregnant women should tackle pregnancy complications. In this regard, they should be strongly advised to proceed with caution:

*“Pregnant women need to get self-care trainings during COVID-19 pandemic ... If they see the signs of pregnancy complications, including spots of blood and sudden bleeding with progressive pain and contraction, headache, blurred vision, heartburn, kidney pain, shortness of breath, and any of the symptoms that are progressive, they should follow the recommended procedures of online pregnancy booklet provided by medical universities until symptoms recover....”* (P6).

To control the COVID-19 infection symptoms, due to the physiological changes in pregnant women’s body, one interviewee emphasized the fact that: 

*“…pregnant women should investigate COVID-19 symptoms in more detail as the hormonal and physical changes in the first trimester and the last trimester may cause similar symptoms such as shortness of breath…. Therefore, they should carefully examine the symptoms and signs, especially if they come along with fever and dry cough.”* (P4).

### 2. Information that Provokes Sensitivity to the Preventive Measures

Information that provokes sensitivity to the preventive measures was defined in three sub-categories of *“increasing the risk perception”*, *“being informed of the requirements of safe midwifery services”*, and *“basic information about the COVID-19 infection”*.

Increasing the risk perception of pregnant mothers depended on the literacy level of pregnant mothers as literacy could increase risk perception. 

*“The literacy level of the pregnant mother is the most important criteria in meeting information needs....The information needs of pregnant women can include general information about acute respiratory infections, routes of transmission, and symptoms ....which should be appropriate for all women with different levels of education...”* (P11).

Risk perception is also concerned with understanding the necessity of limiting close contact with children and relatives. 

*“... Mothers should know how to reduce contact even with close relatives, especially children, because their hands are more likely to be infected because contact with their environment is greater, and as a result, they are more likely to transmit the infection to the pregnant mother ...”* (P3).

Besides, pregnant women need to know the characteristics of safe midwifery services that midwives or gynecologists should provide during the pandemic. Just as health authorities make the medical staff more sensitive to preventive measures for pregnant women, pregnant women also need to be aware of the right and wrong decisions of their midwives and physicians in the field of patient-based and health provider safe actions. Therefore, pregnant women should have information that helps them select a midwife or gynecologist wisely who follows preventive measures in face-to-face visits. The following are statements of the participants about patient-based and health provider safe actions:

*“Pregnant women should be aware of the necessity to cancel pregnancy classes... The emphasis is more on in-person and individual training to obey transmission-based precautions.... and disinfecting blood pressure devices and other medical equipment as much as possible for everyone ... healthcare visits should be provided when there are less gridlock and crowd in the medical buildings... Pregnant women should pay attention to the fact that the health providers halve the number of in-person visits and timing of visits in low-risk pregnant groups....”* (P8)

Finally, having basic information about the COVID-19 infection would also sensitize pregnant women to preventive measures. Therefore, pregnant women should receive the latest information about the vaccination against COVID-19 and epidemiology of the COVID-19 infection. In this regard, the subcategory of *“basic information about the COVID-19”* emerged.

Some interviewees discussed the necessity of awareness about the COVID-19 epidemiology.

*“….pregnant women should receive the latest information about epidemiologic history, transmission route, and incubation period of the COVID-19 infection …”* (P13).

One participant discussed the necessity of making pregnant women aware of the vaccination risk factors: 

*“Pregnant women should be aware that no specific vaccine or medicine has been recommended to treat COVID-19 infection. Thus, they should take wise precautions to prevent the spread of the disease...”* (P15).

### 3. Awareness of the Perceived Threat

Interviewees recommended that pregnant women should be informed of the perceived threats in three sub-categories of *“babies born to mothers with known COVID-19”*, *“risk factor of vulnerability”*, and *“social media”*.

They discussed the ways to control concerns about the health of the fetus/baby born to a mother getting the COVID-19 infections. They recommended the following information needs:

*“Pregnant mothers should know that they should avoid skin contact with the newborn. Therefore, the baby should be quarantined for two weeks.... However, vertical and breast milk transmission has not been proven if mothers get COVID-19 infection....”* (P16).

Since pregnant women are among the vulnerable groups, they need to be aware of the factors that can make them more vulnerable:

*“Pregnant mothers should be more sensitive to chronic diseases that are common in pregnancy, such as diabetes and high blood pressure, etc. ….Pregnant women who have chronic diseases should be aware that the infection would cause more severe complications and make them more sensitive to side effects...”* (P17).

Social media can pose threats to pregnant mothers. In this regard, threats refer to decreasing social trust, disseminating incorrect information, and increasing fear. 

*“... Pregnant women might trust fake social media more than medical staff, so they should be aware of the necessity to stay away from unreliable media (Social trust)…”* (P12).

*“The first advice when a community is in a state of panic caused by social media is to proceed with extreme caution…”* (P18).

### 4. Awareness of the Health System Functions in COVID-19 Pandemic

The last category emphasized the functions of the health system that should be clear to all pregnant women. The functions contain two subcategories: *“being aware of information sources of Medical Universities”* and *“being aware of the services provided.”*

Regarding the first subcategory, awareness of the links of scientific and general information, and visual pieces of training on the Universities’ websites reassure pregnant mothers of access to credible information sources. 

*“….One of the information sources is the scientific articles that have been published in journals. The ministry of health and health centers also provide information to pregnant mothers... and there is some scientific information on the websites of Medical universities such as https://corona.mui.ac.ir / fa and https://arman.vums.c.ir/corona...”* (P14).

Regarding the second subcategory, medical universities re-organized the care management of pregnant women by providing adequate health services, referral health centers, home-based maternal services, and telemedicine services.

Regarding the adequacy of health services, one participant discussed:

*“Pregnant mothers should make sure how their condition will be checked and followed up after discharge... they should be informed of the services that would be provided by health liaison experts who are in charge of following up discharged mothers ...”* (P7).

The interviewees emphasized the importance of informing pregnant mother as to the referral centers:

*“Pregnant women should be aware of the hospitals that are referral health centers for confirmed cases of COVID-19 infection.... The referral hospitals admitting suspected or confirmed cases of pregnant women should be announced to scientific associations and should clarify the public…”* (P12).

Participants emphasized the importance of informing pregnant mothers about available midwifery services at home:

“Pregnant mothers should know that they can apply for a home visit ... In this regard, a midwife or doctor would visit pregnant mothers at home, so that they do not have to go to medical centers...”

Regarding the telemedicine services that pregnant mothers need to be informed of:

*“I think health care providers who have the phone numbers and addresses of the pregnant women can call them and train them in the prevention procedures, signs, and symptoms, and so on over the phone...health providers should get feedback on training, and if pregnant women are faced with some complications during days and nights, they could call and get relevant guidance ...”* (P10).

## DISCUSSION

The results showed that the information needs of pregnant women in the Covid-19 pandemic consists of four main areas: self-efficacy of pregnant women, information that provoke sensitivity to the preventive measures, awareness of the perceived threat, and awareness of the health system functions in COVID-19 pandemic.

The self-efficacy category refers to making pregnant women aware of general and specific self-care principles. Recent studies on COVID-19 focus more on general self-care principals as knowledge acquisition, preventive behaviors, and specifically mental health. ^17 , 18^ However, a research proposed self-efficacy indicators as recognizing symptoms and home-managing of COVID-19 infection that are considered in the “specific self-care principals” subcategory in our study. ^19^ Besides, a study showed that motivating pregnant women in following healthy behaviors would increase their mental health (general self-care principles). Reviews of studies have also shown that it is necessary to make pregnant women aware of proper self-care and routine hygiene procedures. In this regard, during the quarantine period, due to receiving a low dose of vitamin D, taking supplements and consuming nutritious diets are recommended, like others. ^20 , 21^

In order to provoke sensitivity to preventive measures, we recommend increasing the risk perception of pregnant mothers. Previous studies recognized two factors of anxiety and social support in relation to the risk perception of pregnant mothers. ^22 - 24^ In this regard, a study found that, during the COVID-19 pandemic, maternal anxiety would deeply be affected by social support and risk perception. ^25^ However, we found that risk perception could be moderated as to the literacy level of the mothers and awareness of social distance. Regarding the safe midwifery services during the pandemic in Kenya, research recommended some community-based measures such as decreasing the burden of patients’ visits in hospitals and networking community health workers with communities. ^26^ The goal is directing the focus of both patients and health- care providers to the required precautions. Additionally, pregnant women should be equipped with basic information about the COVID-19 infection. Risk of vaccination against COVID-19 infection is one of the most important topics in basic information. In this regard, researchers developed communication guidance for pregnant women about disseminating transparent information and scientific vaccine findings. The researchers showed that the knowledge on the usage of vaccines and the possible side effects is the right of all pregnant women. In addition, as there is so much exaggeration about the harmful effects of medicine and vaccines for pregnant women, the scientific results about the effectiveness and safety of the medicine/vaccines should not just be reported to health authorities. Besides, knowing that the epidemiological finding, like clinical signs, laboratory results, and radiographic criteria, in pregnant women with COVID-19 are similar to other affected adults would reduce the concerns of pregnant women. ^20 , 21 , 27 , 28^ However, being informed of epidemiological studies/data/evidence for pregnant mothers is recommended in our study.

Awareness of the perceived threats posed to the health of babies born to mothers with known COVID-19 is very important. In this regard, there are highly controversial recommendations in articles about the health of an infected mother and the fetus. A research has recommended abortion and termination of pregnancy, amputation of the umbilical cord of the premature infant, separation of the infected mother from her infant, and disruption of breastfeeding. ^29^ Besides, the decreased risk of transmitting the pathogens from an infected mother to the fetus has not yet been proven. ^30 , 31^ In addition, the Centers for Disease Control and Prevention (CDC) have found that the risk of transmission is higher in the 37 weeks and over. ^6^ Other studies also did not show vertical transmission or transmitting through breast milk. Besides, pathogens have not even been detected in the cord blood. ^32 , 33^ However, a research reported premature births or infants with respiratory distress from the mothers infected with COVID-19. Additionally, pregnant women should be aware of the social media threats. ^34^ Therefore, looking for reliable sources of information is very important in social media. The reason is while social media has been known effective in educating and screening high-risk groups, it can lead to public fear and reduce social trust by providing false information. However, some interviewees discussed that the stress of information bombardment has caused some experts to ignore all information related to the pandemic. The consequences of ignoring are dangerous for pregnant women. The phenomenon of ignoring too much information is due to the “self-conflict” resulting from doing what is needed to be done against what the person is willing to do. ^35 , 36^ Therefore, people prefer to ignore what they have to do consciously. In this regard, increasing social trust to information sources of medical universities would be helpful that is to be discussed in the next category. Pregnant women were also high-risk cases as they had shortness of breath during pregnancy and displayed more severe respiratory symptoms in previous epidemics of influenza and SARS. Therefore, clinical manifestations should be reported meticulously. ^37^ The above-mentioned points are presented in the subcategories of “risk factor of vulnerability”. Finally, it is better to explain the factors affecting high-risk pregnancies, such as anatomical and immunity changes in simple language. ^16^

The results showed that most information needs that were overlooked were related to the announcement of the health system functions. Some studies have shown that awareness of information sources of Universities effectively reduces the stress of treatment and prevents the conflicting emotions of family members, using home remedies and traditional medicine, and ensuring as to maternal health. ^38 , 39^ In the subcategory of “being aware of the provided services”, although the state officials emphasized notifying the public of referral health centers for medical management of pregnant women with COVID-19, even some interviewees (especially those working in the private sector) did not have accurate information about COVID-19 referral centers. Besides, in order to give assurance of adequacy of health services, a study emphasizes that pregnant women need to know the follow-up services that the Medical Universities have provided for those who have recovered from COVID-19 infection. On the other hand, due to the coincidence of the peak period of the COIVD-19 epidemic with the Nowruz holiday, officials emphasized home quarantine. ^40^ As a result, the connection of pregnant women with private health centers, doctors’ offices, and mobile midwives became limited or completely lost. Besides, the cancelation of pregnancy preparation classes led to the loss of a credible information source. Therefore, it is essential to inform pregnant women of active public health centers and home-based maternal services to receive emergency midwifery and delivery services.

The limitations of this study are the COVID-19 epidemic conditions in which the home quarantine period coincided with the Nowruz holidays. Therefore, access to the study population, such as midwives and obstetricians, was limited for the researchers. Therefore, the necessary contacts were made through public and private hospitals. Besides, the strength of this study is considering the information needs of pregnant women as the research priority of Isfahan University of Medical Sciences for the COVID-19 pandemic. Therefore, all experts and specialists in obstetrics and midwifery were focused and sensitive in the infodemic research field.

## CONCLUSION

The results have shown that the self-efficacy of pregnant women in the pandemic depends on recognizing general and specific self-care principals. Regarding this, provoking sensitivity of pregnant women to preventive measures can be achieved within deeper risk perception and awareness of the characteristics of safe midwifery services. However, they need to be aware of social media threats and the risks for babies born to mothers with known COVID-19 and vulnerable pregnant women. Regarding the necessity of awareness of the health system functions, approved communication channels by the health authorities should give information to all physicians and pregnant women. Besides, if pregnant women were already aware of the facilities and services provided by the Medical Universities, they would treat more consciously due to reduced stress and worries in dealing with the epidemic. Therefore, if pregnant women are aware of the required information needs, health care providers will ensure that prenatal care is received effectively, and the rate of the mortality of high-risk pregnancies and congenital anomalies will reduce. In this regard, providing formal instructions is specifically for pregnant mothers that are adjusted for all health literacy levels, deeply recommended.
